# The Radical Cure of Hernia, Particularly with Children

**Published:** 1893-01

**Authors:** G. Felizet

**Affiliations:** Surgeon to Tenon Hospital, Paris, France


### Southern Medical Record.

A MONTHLY JOURNAL OF MEDICINE AND SURGERY.

VOL. XXIII. ATLANTA, GA., JANUARY, 1893. No. 1

### Original Articles.

### THE RADICAL CURE OF HERNIA, PARTICULARLY WITH CHILDREN.

BY DR. G. FELIZET, SURGEON TO TENON HOSPITAL, PARIS, FRANCE.

Translated by THOS. H. MANLEY, A. M., M. D., Visiting Surgeon to Harlem Hospital, New York.

(*Concluded.*)

The testicle being discovered, was drawn out. It seemed to be wanting in a vaginal investment. By traction of the testicle with a forceps and the finger, a conical expansion of a rucumbrance was seen to rise from the circumference of the ring, in which could be seen the rudiment of a sac, the vas-deferens and the spermatic vessels. It was dissected from its connection as freely as possible and opened near its apex. The finger now introduced recognized the intestine free from all adhesions, which was easily reduced.

The sac was ligated and divided. The testis manifested a marked tendency to remount. The pillars, now that the testicle was outside the ring, were carefully sutured. Four catgut sutures were employed, they being so inserted as not to unduly compress the cord. The testicle in its new position ested against the sealed external ring.

In six days, without accident, union of the incision was complete. There has been no more trouble from symptoms of strangulation. A truss was applied with its bulb so constructed in its inferior concavity as to effectually press the testis downward. The contraction of the crucial incision during cicatrization served very effectively in maintaining the testis in its new position. But it manifested a decided tendency to return every time as soon as the truss was removed. When the patient returned home, he was directed to wear the truss night and day. The child was operated on January 25th, 1890. He returned to us on the 8th of July, same year. He had had no more suffering through hernial complications. Not a trace of the hernia remained. The testicle is descended two centimetres below the horizontal line and rested on the base of the scrotum, even when the truss is removed. It was then as large as the opposite organ. The sutures of the pillars had served a most useful purpose.

Sometimes, at the inferior part, there exists an excavation, in which the testis is inclined to rest with a tendency to return into the belly; but we are persuaded that this would not so frequently occur if we employed silk, silver or gold suture. It is incontestible, however, that in many cases the pillar sutures serve a most useful purpose 1st: to maintain the testicle outside. 2nd: to temporarily close the portal and prevent the protrusion of the intestine.

The transverse incision has its advantage in those cases of ectopic testes particularly, as permitted us to more readily close the ring and cut off communications with the abdominal cavity. Certainly, these cases are exceptional, and in general the longitudinal incision suffices in ordinary practice, unless we desire to approximate the pillars.

### CONSECUTIVE PRECAUTIONS AFTER THE OPERATION FOR THE RADICAL CURE OF HERNIA.

When cure is accomplished, the wound is free from any discharge; swelling and pain have disappeared over the zone of operation, but we must yet require our patient to wear, for a time, a protective dressing: Salicylic powder, bismuth or amidon sprinkled over a pad. This will be renewed daily.

The patient keeps the bed for twenty days or more. Barker and McEurn speak of six weeks, and nothing would be better if we could obtain this sacrifice. It is really a sacrifice.

The world has a tendency to believe that the radical cure is an immediate consequence of the operation. We can scarcely wonder at this, as the fault is rather with surgeons, who in retaining the word radical cure, seem to encourage the delusion.

The term, radical cure for hernia, would seem to us much the most judicious and certainly the most true. We operate, nature cures. We certainly have a right to say this, because many of the insuccesses which we hear of with complaisance, are cases in which bodily movements were made too early, and as a consequence of this complaisance, the want of concord between the surgeon's promises and the patient's hopes are often only too apparent.

The patients must not attempt to walk about too early, but should maintain the recumbent posture as late as possible, before they return to their occupation, particularly if it is such as entails heavy labor.

With many young children, prolonged repose is perhaps more indicated than with adults, by reason of their docity and incessant movements. It may be necessary with them sometimes to employ the bromides for the purpose of securing the necessary calm. We must, above all things, guard them against colds or bronchitis, as the most vicious of all are efforts at coughing. The digestive organs should be kept in good condition.

We may naturally expect some constipation in consequence of prolonged rest in bed. Accordingly we may have recourse to small doses of calomel and the tincture of nux vomica. We should not, as ordinarily in those conditions, have resort to leguminous food, greens or fruit to keep the bowels open, as they all cause an evolution of gas which greatly distends the intestine.

Tincture of nux vomica acts on the muscular fibre of the intestine, which it tones and strengthens. The diet should consist of toast and fish with other non-stimulating articles of food. All can understand the importance of a careful nurse under these circumstances, as when the wound is freshly closed and all swelling has vanished, we must for a time prevent all unnecessary lesion, as all tendency to impulsion has not yet been removed.

It is now with nature to complete the work which the surgeon has but prepared. This it will usually accomplish by the organization of the tissues; by their adhesions to the walls of the track; a definite thickening of the flat septum of the peritoneum; absorb the ligatures, if of catgut; in a word, to return things to a normal state. The object is really attained when the point, feeble at one time, has been strengthened by the installation of solid material over its site. When this is properly achieved, we can understand that it is not the work of a few days.

When, after a reasonable time, on a most minute examination, no trace of a hernia is discovered, as a precaution against return, should his patient be made to wear a truss? Surgeons are divided on this point.

Mitchel, Banks, Lucas-Chappionniere and Legond recommend its usage; while others, notably Thiriar, Socin and Stokes, reject it and accuse it of stretching and thining the cicatrix; of being useless and adding nothing to the cure, which must, by its definition, be radical. The latter claim that it would be playing with words to say that a cure is radical, which demands for its continuance the use of the truss.

The truss, however, like the temporary or prolonged dry dressings, the dorsal decubitus, absence of straining and hygiene of the intestine, is a useful precaution which we regard as of great importance, for considerable time after the operation. During the long period of reparitive organization, which follows the operation for cure, the hernial region should be sustained by a support which will in no way prejudice final success.

If, as has been said, the truss attenuate the cicatrization, then the original cicatrix was of no use, or else the truss is badly constructed and unfit to use. In the latter case we must change the truss. We do not believe that it is necessary to have recourse to any special truss, as they all are usually efficient if they give ample support and occasion no pain. The French and English trusses are equally efficient.

When the apparatus is applied, we should have the bulb comfortably adapted with a lage flat surface, which will exercise gentle pressure and not become displaced. M. Lucas-Chappionniere has invented a model, which has been very satisfactory with me. It has a large bulb fixed to the umbilical spring of Dobleau and retained by a band through which passes a leather strap. It is the France-Comtoise, spoken of by Malgaigue, "a truss in which the strap is soft and the end of the spring placed through the center of the bulb."

M. Berger, whose experience is considerable in those matters, has not been able "to see any advantage which the truss presents over those made according to measure, provided with a flat, suple spring and less voluminous than that conceived of by our distinguished confrere. The substantial use of the truss, after many operations, seem to me incontestible. The question is to know how long and when it is necessary to wear it.

Here the question of age is of prime importance. If the patient has passed the fortieth year, has been operated on for a large hernia, which existed for a long time, with a large neck to the sac, the person in mediocre health, there is no illusion about it, the truss must be worn almost indefinitely. In a young man of firm, full muscle, the truss is necessary during the period of tissue organization, of which we have spoken, perhaps for some months, sometimes oftener, when one's occupation is laborious and there is a tendency to relapse.

With youths from 12 to 14 years old, the time required to wear the truss is much less. Less yet, between 8 and 12, and it need not be used at all in those under 4 years of age. Under four years, we think, in the event of the complication of bronchitis or whooping-cough, presumably a truss serves no useful purpose and nothing is more instructive and convincing on this point than to examine the hernial region on a young subject suffering from these maladies. The tumefaction of the cellular tissues persists a long time and forms a sort of adherent barricade to the wall of the track, continuing with the tumefaction of the cord as far downwards as the root of the testis.

This plastic mass is slow to be absorbed. It plays the role of the ideal bulb (pelote), fixed, flat and indolent. Thanks to it, the truss is useless in the first years of life if the child does not cough much and is well developed. If the young patient emaciates, and above all, if he has whooping-cough or bronchitis, it may be prudent to wear a small truss, which will afford ample protection.

But these are points which will appeal to the judgment of each surgeon to decide according to circumstances. What we wish to emphasize is, that the use of the truss is but one of the precautions to be observed while the reparative work of nature is progressing, and that the necessity and duration of its employment are subordinate to conditions, unimportant or vervins, in which this definite repair, the veritable radical cure is accomplished.

THE DOUBLE OPERATION FOR THE RADICAL CURE IN AN INFANT EIGHT MONTHS OLD.

This observation here presents other interests than those attaching to a surgical rarity. It proves the possibility of performing not once but twice, an operation reputed to be long and difficult on a very young infant, the youngest which has submitted to this operation up to this time.*

M. D., born September 29th, 1889. He was brought into Tenon Hospital, June 6th, 1890. The infant was born at term and was well formed. The mother was startled shortly after its birth when she saw its voluminous scrotum, but she said nothing to the medical attendant about it. The child was about six weeks old, when one day while it was crying she saw the large hernial mass for the first time.

She alleged that both sides seemed equally large. Her two oldest children had no hernia. The father was equally indemnified and his ancestry, but the maternal grandfather had a descent.

---

*Dr. Felizet operated on the 6th of June, 1890. I operated on a baby for the radical cure of hernia, three weeks old, for Dr. J. B. Reade, March 2nd, 1890.—*Trans.*

A small caout-chouc truss was then applied, but the hernia slipped under it, with the effect of provoking colicy pains. The different trusses were successfully tried but without result, as far as the retention of the hernia was concerned. The protrusions had more than doubled their original volume, beside colic and vomiting now were almost incessant.

The baby was now examined by many surgeons, who declared that the operation for radical cure was certainly indicated, but there must be delay 'till the child was at least 3 or 4 years old. The hernia had never been the seat of accident. Taxis is easy when the hernia is down a long time and the child is in repose. It is difficult, however, of reduction when the infant cries or strains.

What particularly worried the parents, was the increasing volume and rapid augmentation of the size of the scrotum. On examination, we found the scrotum full and distended by a mass of intestine, which extended to the intertesticular line, between the knees and hips. The protrusion on each side is of about the same volume. The reduction of both was easy, but it would not remain more than five or six minutes.

It was quite evident when the baby cried that the herniæ were almost wholly intestine. The two rings are large, quite easily admitting the point of the index finger. Their borders give a distinct impression of elasticity. After reduction, at the base of the scrotum, both testes could be easily felt. They are of normal volume and did not seem unduly sensitive.

The volume of these herniæ was enormous, their development rapid and progressing, the irritability of their retention, the impossibility of wearing a bandage, the frequent fear of accident, colic and obstruction, threatened a strangulation, which one day or another would render necessary a bloody operation. All these reasons seemed to impose an obligation in spite of the extremely tender age of our little patient.

The infant, having been purged the preceding evening, was bathed in the morning and received a simple liniment. On June 9th, 1890, we operated on the right side, with the assistance of Mess. Percher and Camescasse, internes, and Dr.

Butin, externe. We selected the right side because it seemed the side best adapted for the radical operation.

We made an incision five centimeters below the external orifice of the inguinal track, and along the anterior surface of the scrotum. Fine arterioles were ligated in the cellular tissues. They were the only vessels ligated during the operation. With a few light strokes of the scalpel, the sac was reached. As it was well distended, it was readily recognized.

Gradually detaching the sac with the aid of the grasping-forceps, an opening was made in it and the borders separated by the forceps. The index finger was now introduced into the pocket and his loops of the prolapsed intestine were easily reduced. On exploring the declevity of the sac, the bare, serous covering of the testis was felt. It was then a congenital hernia.

At the aperature of the sac, its neck was found to be very elastic. The balloon was now introduced, its small extremity engaging at the terminus of the inguinal track. Occlusion of the opening made in the sac was effected with forceps. Distension was made with the insufflator. The operation was performed, as heretofore described, slowly without the loss of a drop of blood.

Dissection by isolation commenced posteriorly to the elements of the cord, which lay stretched posterior-internal to the sac. Careful dissection of many thin delicate membranes soon led to the red caout-chouc, which appeared like the veritable pellicle, distended. Enucleuatur was continued until the external orifice of the inguinal tract was reached. Demi-tortion and traction brought the sac well down.

A double ligature crossed as high as possible of No. 1 catgut was now applied. The pedicle was now divided from four to five millimetres below the ligature. The stump remounted quickly and disappeared in the abdomen. The sac was partly excised, but that part in which was lodged the testes was not disturbed. The part excised represented the sixth part of the tunica-vaginalis. It weighed twenty centigrammes.

The integuments were now washed with Van Levietin's liquid, iodiform and drainage tube. Six linen sutures were introduced into the skin. The operation, from the commencement of etherization until completion of the dressings, occupied twenty-five minutes. The ultimate results were favorable, but not immediately simple. In the evening the temperature attained 38 degrees (C.), the infant was depressed and agitated. During the night there was no sleep. At no time was there tympanitis, pain, nausea or vomiting.

On June 10th, feebleness continued and the infant is sleepy, temperature 38.5 degs. in the morning, 39 degrees in the evening. Diet, champaigne.

June 11th, an abundant stool. Temperature 31 in the morning, 38.3 degs. in the evenings.

12th, 13th and 14th, diarrhœa. Suppression of the milk, stimulant and champaigne. From this time improvement was continuous; the infant was gay and temperature normal; the general constitution excellent.

June 18th, the dressings were changed; union by first intention is perfect; the sutures above and below were divided. A small opening in the incision was discovered, which was treated with iodine and dry salol.

June 24, cicatrization is complete; the lower aperture is dry and we are preparing to operate on the left side in a few days. The accidents which followed the first operation; the elevation of temperature, agitation, insomnia and profound depression of vital force, seemed to depend on a light iodoformic intoxication. The quantity of iodoform used was small, but he had committed the fault to powder the bare surface of the wound before suturing.

The operation for the radical cure on the left side was practiced on the 9th of July, after the same preparations and same procedure. It was more difficult and occupied forty minutes. Among the causes for the difference we might mention the following:

The hernia on the right side was congenital, and on its internal surface we found the elements of the cord. We had no reason to believe that the left hernia was not all free. Now, this makes a great difference. Our first incision of the bistoury aimed to bear forward and on the sides of a large vascular area, from which it was necessary to separate the elements of the cord, before it was deemed prudent to attack the sac; the first disappointment.

The sac was opened and the lips fixed in the wound with the forceps, but we had the greatest difficulty in trying to return the intestine. Not that the reduction was difficult, but the sac contained three coils of intestine. The intestine was tender, nervous, rebellious and defiant; second disappointment.

It was reduced nevertheless. Before introducing the balloon, the finger explored the sac, and the testis was found with its own proper, independent investment. This presented the characters, anatomically, of the acquired. The balloon was introduced and opening closed. Distention operated with great facility and the operation was quickly concluded.

This operation as has been seen was long, and as the dissection was laborious and difficult, serious precautions were imposed. The disappointment in not finding the congenital condition as in the preceding case was the occasion of requiring more time to manipulate. Iodoform was excluded this time from the dressing. The wound was washed with the antiseptic solution, dusted with powder of salol and salicylate.

The result was most gratifying, and the infant never ceased to be gay. The temperature never went beyond 37.8 degs. The next day the little one had a free motion, and the bowels continued regular thereafter.

On the 23rd of June, the infant was taken from the hospital entirely well. The scrotum is moderately distended. Before the rings we can feel two distinct masses, fixed, hard and indolent, which transmit no impulse during coughing.

The infant was brought to us September 4th. He had suffered from a severe cold. The scrotum was normal. The induration had disappeared. One cannot feel the external orifices of the inguinal tracts. No trace of impulse on crying or coughing. Because of his bronchitis we advised the temporary use of a small truss.

September 25, he was returned to us, yet coughing, and during the night with a strain much like whooping-cough. But in spite of all this, the local state is absolutely irreproachable. The radical cure had resisted effectually, effort of every description.

